# Supplementary material for: Fabrication of Enhanced Mechanical Properties and Intrinsic Flame-Retardant Polyurethane Elastomer Containing 4-(Phenylethynyl) Di(Ethylene Glycol) Phthalate
Source: Polymers (Basel). 2021 Jul 21;13(15):2388. doi: 10.3390/polym13152388 (PMC8348043; doi:10.3390/polym13152388)
Supplement: Supplementary file 1 [file polymers-13-02388-s001.zip › polymers-1285319-supplementary.pdf]

Electronic Supporting Information

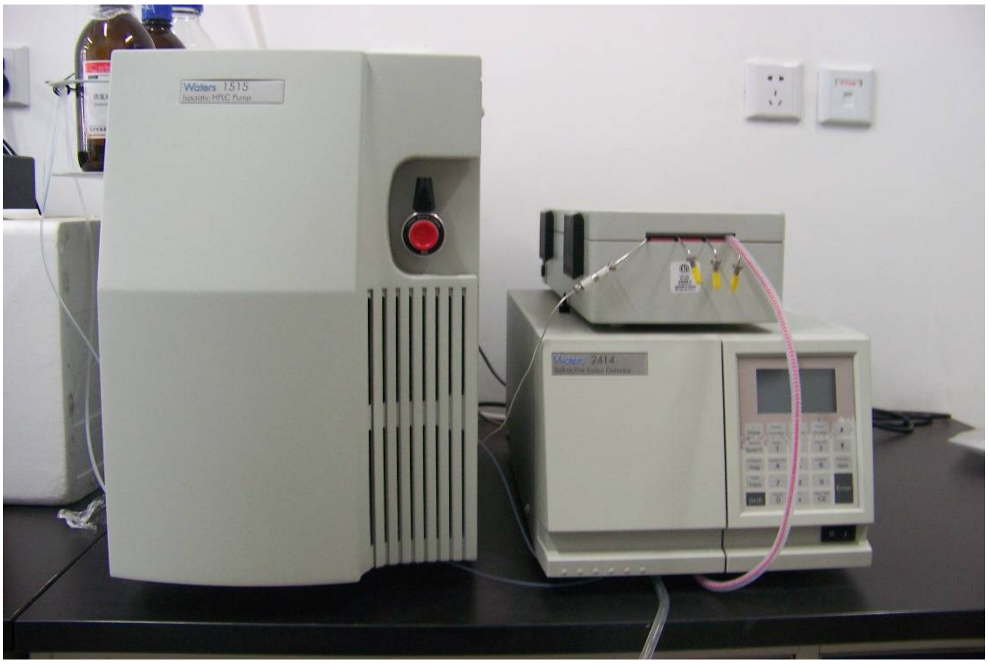

Figure S1. Digital photograph of the equipment for testing molecular weight of PEPE

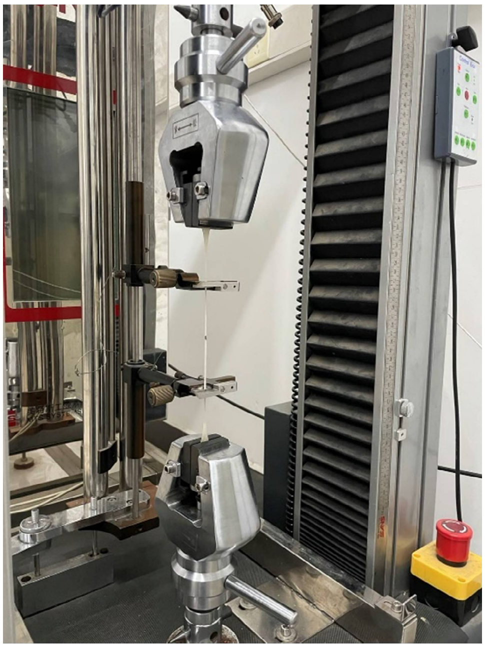

Figure S2. Digital photograph of tensile test process containing 1.35%PEPE-TPU

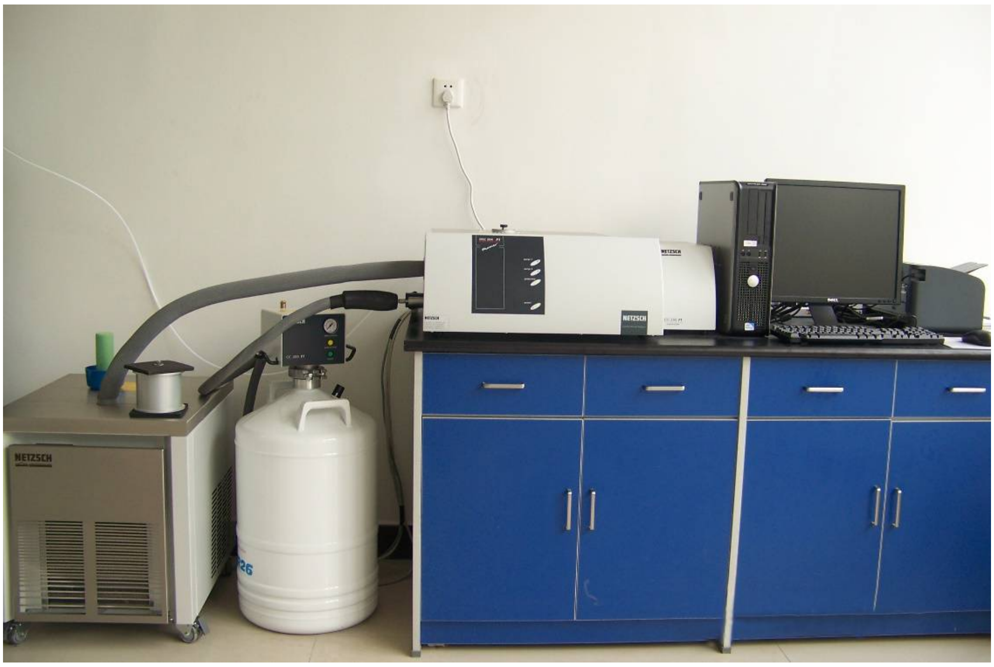

Figure S3. Digital photograph of the equipment of thermogravimetry analysis test

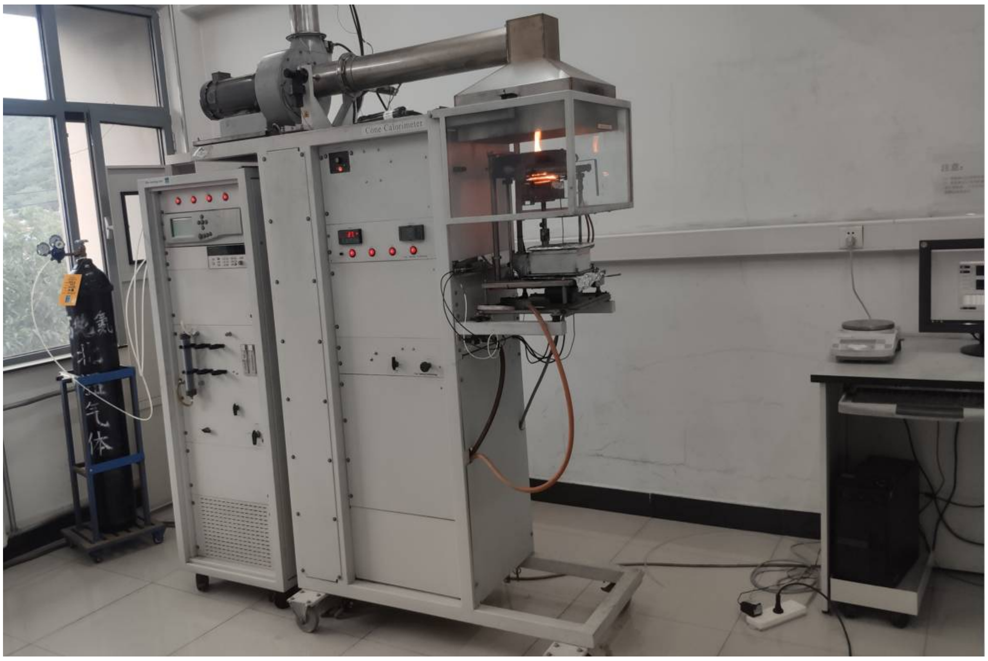

Figure S4. Digital photograph of cone calorimeter test process of TPU with PEPE

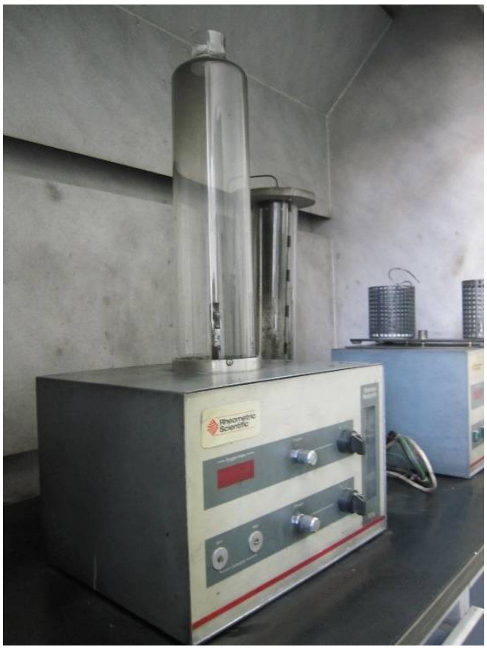

Figure S5. Digital photographs of the equipment of limiting oxygen index test
